# Prevalence and comparisons of alcohol, candy, energy drink, snack, soda, and restaurant brand and product marketing on Twitch, Facebook Gaming and YouTube Gaming

**DOI:** 10.1017/S1368980021004420

**Published:** 2022-01

**Authors:** Caitlyn G Edwards, Catherine C Pollack, Sara J Pritschet, Keally Haushalter, John W Long, Travis D Masterson

**Affiliations:** 1 Department of Nutritional Sciences, The Pennsylvania State University, State College, PA 16802, USA; 2 Department of Biomedical Data Science, Geisel School of Medicine at Dartmouth College, Hanover, NH, USA; 3 Department of Epidemiology, Geisel School of Medicine at Dartmouth College, Hanover, NH, USA

**Keywords:** Food marketing, Twitch, Streaming, Influencers, Energy drinks

## Abstract

**Objective:**

To compare and evaluate the prevalence of food and beverage marketing on the livestreaming platforms Twitch, Facebook Gaming and YouTube Gaming, as well as examine growth of food and beverage marketing on these platforms over a 17-month period of data collection.

**Design:**

Cross-sectional data were analysed across three livestreaming platforms and six food and beverage categories: alcohol, candy, energy drinks, snacks, sodas and restaurants.

**Setting:**

Stream titles of livestreamed events as well as corresponding hours watched on Twitch, Facebook Gaming and YouTube Gaming.

**Participants:**

None.

**Results:**

There were significant differences between the use of food and beverage brand mentions in stream titles across all three studied platforms (*P* < 0·05), as well as hours watched across platforms (*P* < 0·05). Energy drinks dominated food and beverage brand mentions across platforms, followed by restaurants, soda and snacks. All platforms demonstrated growth over the 17-month data collection period. Post-hoc analyses revealed that the COVID-19 pandemic impacted both immediate and sustained growth across all platforms, with the greatest impact observed on the Twitch platform.

**Conclusions:**

Food and beverage marketing as measured through stream titles is widely prevalent across the three most popular livestreaming platforms, particularly for energy drinks. Food marketing on these platforms experienced growth over the past 17 months which was accelerated substantially by the COVID-19 pandemic. Future work should assess the sustained impact this growth may have on marketing practices and eating behaviour.

Rates of overweight and obesity continue to rise worldwide^(1)^. The underlying mechanism that contributes to weight gain is the consumption of energy in excess of the body’s homoeostatic needs^(2)^. Excess consumption of energy can be driven by a number of factors, one of which includes the endemic presence of marketing for food and beverages in everyday life^(3)^. Food marketing and advertising has long been omnipresent and has been shown to impact both the conscious and unconscious mechanisms that regulate food consumption and choice^(4,5)^. Recent and rapid advancements in technology and shifts in entertainment platforms have contributed to the development of innovative marketing techniques, exacerbating the impacts of the so-called ‘obesogenic environment’^(6–8)^. Therefore, it is vital that the prevalence of food marketing and the techniques employed by food companies on new platforms, such as social media, are monitored to understand the reach and potential impact on food choice and eating behaviour.

‘Livestreaming’ is a relatively new form of social media entertainment that has seen a dramatic climb in popularity over recent years^(9,10)^. Currently, the three largest livestreaming social media platforms in the USA are Twitch (owned by Amazon), YouTube Gaming (owned by Google) and Facebook Gaming (owned by Facebook)^(11)^. All three platforms enable streamers to broadcast live audiovisual content to viewers. In addition to the live broadcast content, streamers are able to directly interact with their audiences in real time. This is accomplished through a live chat feature where viewers of the streaming content can send messages to both the streamer and other viewers, allowing for the community to interact in an organic way. Streamers can also create a profile page which is displayed below their stream where they can provide information to their audiences about their content. This page often includes persistent product advertisements and endorsements^(6)^. Through an advertisement-based revenue model, streamers will often incorporate sponsored products into their entertainment content through a variety of means including overlay ads, product placements, branded mini-games and giveaways. In this way, streamers can take on the role of ‘influencers’ or individuals with the ability to influence potential buyers of a product or service by promoting or recommending items on social media. This influencer-led advertising content is then presented alongside more traditional website-driven content such as pre- and mid-roll video and banner ads. Advertising on livestreaming platforms therefore combines a number of social media marketing techniques in an overlapping and interactive manner, which is unlike many other social media and entertainment platforms. According to the Reactivity to Embedded Food Cues in Advertising Model, this integration of multiple marketing techniques can influence the advertising effect process^(12)^. The high levels of integration of marketed content on livestreaming platforms may result in less cognitive elaboration and less engagement of consumer defences such as persuasion, knowledge and scepticism, leading to a higher persuasive impact of the marketing strategy^(12)^. While this remains to be thoroughly validated in a livestreaming environment, it has been shown that this integration of passive and participatory techniques has proven to be rewarding, as viewing of livestream events has been shown to be motivated by cognitive, affective, personal integrative, social integrative and tension release motivations^(13)^.

While livestreaming is commonly associated with video gaming, its reach extends far beyond the gaming niche. For example, while the most widely used livestreaming platform Twitch is known for its video game broadcasts, it has expanded its reach to include events such as sports, political rallies and music, among others^(14)^. Since its launch in 2011, Twitch has become the most widely used livestreaming platforms^(11)^. Twitch reports an average viewership of over 2·5 million users at any given moment, 30 million average daily viewers and over 1 trillion min of watched content in 2020^(15)^. On a smaller scale, YouTube Gaming reported an average of 871 000 viewers streaming at any time during the fourth quarter of 2020 and over 10 billion hours of livestreaming content in 2020^(16)^. Facebook Gaming saw a 96 % increase from 2019 in average concurrent viewership to 408 000 viewers by the end of 2020, and doubled its hours of streaming content from 0·5 billion at the end of 2019 to over 1 billion by the end of 2020^(17)^. This growth has not gone unnoticed by marketing agencies, including those marketing nutrient-poor, energy-dense foods. Previous work identified energy drinks, coffees and teas as the most widely advertised products on Twitch over an 18-month period^(6)^. However, restaurants and food delivery services and sugar-sweetened beverages were most frequently mentioned in chat messages, even though the marketing of these products through streamers was significantly lower than energy drink products. Despite these variations, all products experienced significant growth in exposure over the study period, including sugar-sweetened beverages and candies^(6)^.

Livestreaming services have produced a profound shift in the media landscape, and content creation has shifted from large corporations and organisations to users as content creators, influencers and brand ‘affiliates’^(18,19)^. Influencer marketing has proven effective on other internet platforms (e.g. YouTube, Instagram), and marketing of unhealthy snacks to children via influencers in particular has been associated with increased food intake even when the influencer discloses that they are advertising a product^(20,21)^. This marketing is supported by the platforms themselves. As an example, in 2020 Twitch introduced a large number of new influencer-centred marketing techniques to increase revenue on the platform. This included the creation of the ‘Twitch Affiliate Program’ to help facilitate contact between brands and streamers^(22)^ and the requirement that streamers run a set number of ads per hour of content^(23)^. Alongside these internal changes, as Twitch is a US-based company, the Federal Trade Commission requires all social media influencers, including streamers, to include ‘#ad’ in the title of a stream when receiving compensation for mentioning a product, regardless of where in the world a streamer may be operating from^(24)^. However, companies have been looking for ways to subvert regulations and partnerships to extend their advertising in unique ways, some more effective than others. For example, a recent Burger King campaign targeted popular streamers and their communities by donating the price of a Burger King meal to a pre-specified group of streamers during one of their streaming sessions. The donation triggered an automated bot to interrupt the stream with a message instructing the streamer and viewers to go to the nearest Burger King and purchase a meal. This type of viral marketing effectively sidestepped #ad disclosure requirements from the Federal Trade Commission while also implying that the streamer was serving in an influencer capacity for Burger King when no such agreement had been made. This campaign received pushback from many of Twitch’s top users and streamers who said such marketing ploys were taking advantage of Twitch donation features to avoid paying streamers as formal influencers^(25)^.

Our group has published previously on the marketing techniques and presence of brand exposure for food and beverage items across stream titles, streamer profiles and chat messages on the livestreaming platform Twitch^(6)^. The current study expands upon our previously published work to describe the presence of and potential differences between food and beverage brand mentions across three major livestreaming sites (Twitch, YouTube Gaming and Facebook Gaming). To accomplish this, we examined brand presence across six distinct categories (i.e. alcohol, candy, energy drinks, processed snacks, sodas and restaurants) during a 17-month time period (i.e. July 2019–November 2020). In addition, we sought to examine the impact the COVID-19 pandemic (i.e. March 2020–November 2020) had on food advertising on these platforms, as it has been reported that advertising budgets shifted significantly to online platforms during this time period^(26)^. This will provide policy makers and researchers an updated understanding on the continued presence and potential growth of food and beverage brand exposure across livestreaming platforms.

## Methods

### Generation of brand and product search term

A list of brands and products was compiled prior to data collection by the research team. The brand list was based on our previously published work^(6)^ but was expanded to include new brands or brands that may have not been advertised on livestreaming platforms previously. To identify new brands, we first consulted updated reports of food marketing trends and practices from the Rudd Center for Food Policy and Obesity^(27)^. We then had two members of the research team examine the profile pages and stream titles of the top 100 worldwide Twitch, YouTube Gaming and Facebook Gaming streamers for current food and beverage brands, both US-based and international, providing sponsorship or being advertised on these platforms^(28)^. Brands that were identified in either the Rudd Center reports or the profile pages and stream titles of the top 100 worldwide streamers were then added to expand our original list of brands. As in our previous work, only core brand names and products were searched, and variants of these brand names were not searched (e.g. ‘Coke’ was searched while ‘Coke Zero’ was not, as the base word ‘Coke’ would still capture this product variant). To account for common misspellings (e.g. ‘Gfuel’ *v*. '‘G fuel’), grammatical errors (e.g. ‘Reese’s *v*. Reeses’), the use of ‘z’ instead of ‘s’ (e.g. ‘Cheez-Its’ *v*. ‘Chees-Its) and general Internet slang (e.g. ‘Chick-fil-A’ *v*. ‘Chikfila’), the finalised list was subjected to an algorithm to generate a variety of spelling variants for each brand^(29)^. In total, 854 search terms were generated (including alternative spellings) to search for 312 brands across six distinct 'pre-identified categories: alcohol, candy, energy drinks (and other caffeinated beverages), processed snacks (foods designed to be eaten outside of meal occasions including: chips, crackers and snack bars), soda (and other sugar-sweetened beverages) and restaurants (and food delivery services that are not restaurant specific). A full list of initial candidate terms is available in the online supplementary material.

### Data collection

All data were extracted from the analytics platform Stream Hatchet © (Stream Hatchet SLU)^(30)^. While our previous work was able to cite three metrics of advertising in the livestream environment (i.e. the stream titles of each individual stream, the profile page of each streamer and the chat room logs) limitations have since been placed on publicly available data that can be extracted from the application programming interface by the livestreaming platforms; therefore, it was only possible to access information for stream titles. Additionally, as Twitch was the only platform for which we would have been able to capture chat room data, we chose not to examine it for the purposes of this comparative analysis. Brand prevalence in stream titles and hours of viewership of stream titles were obtained using a Python 3 script that implemented the Selenium library to interact with the Stream Hatchet graphical user interface and automatically and iteratively search each brand and product. Data were collected in December of 2020 for a time period from July 2019 through November 2020. All mentions of food and beverage brands in stream titles were extracted, not just titles containing #ad, as brand exposure was still a result of use of the food and beverage brand, whether sponsored or not.

### Data analysis and post-processing

All analyses were conducted using the R language and environment for statistical computing (version 4.0.3, R Studio Team, 2020) using the RStudio Graphical User Interface. All data mining was conducted in the Spyder integrated development environment with Python 3 (version 3.8.0). All code used in this project is openly available at https://osf.io/gm3wd.

To assess brand exposure, we utilised two metrics: stream titles and hours watched. Stream titles refer to the unique mention of a brand or product name in the name or ‘title’ of the stream. This stream title appears below the livestream content and is visible throughout the streaming experience. The number of unique titles containing each brand or product was quantified and referred to as ‘titles’ throughout. Hours watched (referred to as ‘hours watched’ throughout) was calculated as: *h*_*total*_ = *h*_*v1*_ *+ h*_*v2*_ *+ h*_*vn*_, where *h*_*total*_ denotes the total hours of content watched and *h*_*vn*_ denotes the hours watched per unique viewer. Therefore, the hours watched is a sum of all the time unique viewers spent viewing the livestream content. In addition, because of the *a priori* knowledge of differences in overall viewership numbers on the three livestreaming platforms of interest^(11)^ (i.e. larger viewership is found on Twitch than the other examined platforms), the ratio of hours watched per title was calculated to standardise potential differences in exposure (i.e. hours watched) to food and beverage products/brands in stream titles across categories and platforms on a monthly basis. The total number of unique titles and total number of hours watched under each title were assessed on a monthly level for each brand in order to examine trends over time. Aggregated monthly totals were collapsed to a total value in order to examine differences between platforms over the study period.

The distribution of titles and hours watched by brand category and across platforms was described using frequencies and differences were examined using *χ*^2^ tests. To assess the change in prevalence of brands in both number of unique titles and the number of hours watched over time, linear regression analyses were conducted for each category and for each platform. In these linear analyses, dependent variables were titles or hours, and months (as a discrete variable) were considered independent variables. To evaluate the individual contribution of specific brand titles to the overall brand category, the top five brands in each category were identified for each platform and their percentage contribution to the total stream titles or hours watched was calculated.

In addition to the main analyses, we conducted post-hoc analyses to examine differences in linear growth trends in the data pre- (i.e. July 2019–February 2020) and peri- (i.e. March 2020–November 2020) the COVID-19 pandemic. This was done using interrupted time series analysis with March of 2020 as a breakpoint to analyse the differences. This analysis evaluates the linear trend pre-March, the immediate change in exposure from March to April and the linear trend post-March. Prior to running any statistical tests, a significance level of *α* = 0·05 was chosen.

## Results

### Analysis of all platforms

**Fig. 1:**
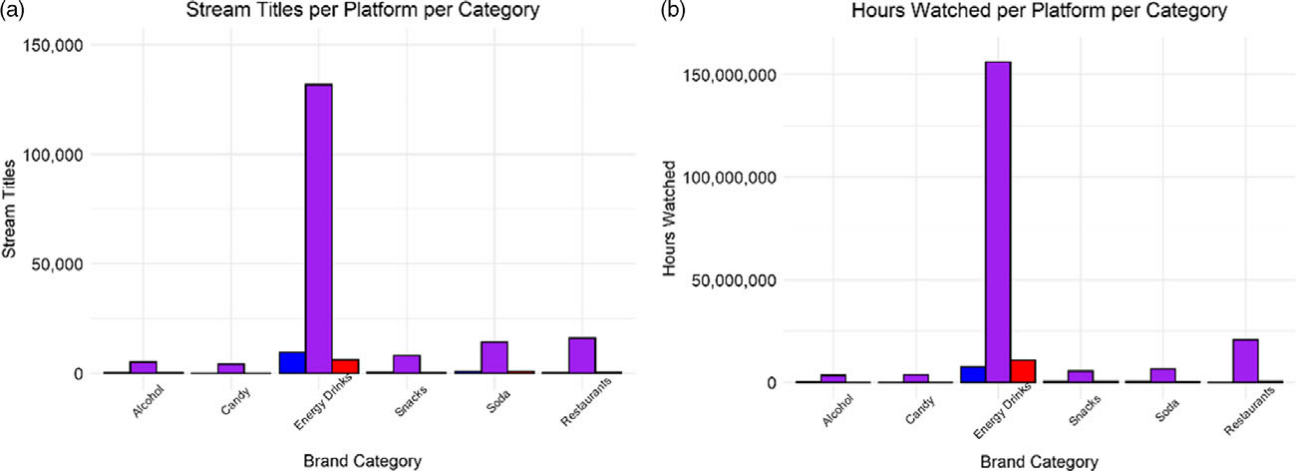
(a) Bar plot depicting the number of stream titles containing a food brand or product name for all platforms (Twitch, Facebook Gaming, YouTube Gaming) across six food or beverage categories (alcohol, candy, energy drinks, snacks, soda, restaurants). (b) Bar plot depicting the hours watched for all platforms across categories. 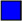, Facebook; 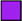, Twitch; 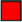, YouTube

Table 1 displays the top five brands mentioned in stream titles across platforms and categories, as well as the contribution that each brand makes to the total stream titles in each category. There was a significant difference in the number of food and beverage brands from any category mentioned in stream titles (*χ*^2^ = 900·7, df = 10, *P* < 0·001) between livestreaming platforms (see Fig. 1). Across all stream titles observed, Twitch was responsible for 90 % of all food and beverage brand mentions in stream titles, while Facebook contributed 6 % and YouTube contributed 4 %. Energy drinks were mentioned the most frequently in stream titles across all three platforms (74 %), followed by restaurants (9 %), soda (8 %), processed snacks (5 %), alcohol (3 %) and candy (2 %). In terms of hours watched, there was similarly a significant difference in the hours watched under unique titles containing food and beverage brands (*χ*^2^ = 2 068 296, df = 10, *P* < 0·001) between livestreaming platforms (see Fig. 1). Twitch was responsible for 90 % of the total number of hours watched, while YouTube Gaming contributed 6 % and Facebook Gaming contributed 4 %. The product category with the most hours watched was energy drinks (80 %) followed by restaurants (10 %), soda (3 %), processed snacks (3 %), alcohol (2 %) and candy (2 %).

**Table 1:** Top five brands in each of the six brand and product categories on Twitch, Facebook live and YouTube Gaming

Category	Twitch					Facebook Gaming					YouTube Gaming				
	Brands	Titles	%	Hours	%	Brands	Titles	%	Hours	%	Brands	Titles	%	Hours	%
Alcohol (*n* 56)	Total	5180		3 443 472		Total	202		188 065		Total	186		119 243	
	Top 5	3591	69	2 878 281	84	Top 5	160	79	176 768	94	Top 5	139	75	107 824	90
	White Claw	1232	24	288 070	8	Modelo	57	28	61 614	33	Jack Daniels	49	26	5239	4
	High Noon	714	14	364 099	11	Heineken	55	27	104 090	55	Guinness	43	23	87 076	73
	Budweiser	679	13	2 004 709	58	High Noon	31	15	8045	4	High Noon	19	10	9324	8
	Modelo	555	11	160 828	5	Crown Royal	9	4	557	0	Budweiser	14	8	4320	4
	Heineken	411	8	60 575	2	White Claw	8	4	2462	1	Modelo	14	8	1865	2
Candy (*n* 25)	Total	4249		3 897 854		Total	117		36 761		Total	153		141 040	
	Top 5	2707	64	2 728 254	70	Top 5	98	84	21 369	58	Top 5	121	79	66 617	47
	Oreo	975	23	196 368	5	Oreo	56	48	8708	24	Oreo	46	30	36 575	26
	Snickers	601	14	1 266 912	33	Kit Kat	22	19	7060	19	Kit Kat	28	18	22 823	16
	Skittles	572	13	178 690	5	Skittles	8	7	1986	5	Milky Way	18	12	3018	2
	Reese’s	294	7	1 011 653	26	Milky Way	8	7	2952	8	Twix	17	11	3884	3
	Twix	265	6	74 631	2	Snickers	4	3	663	2	Nestle	12	8	317	0
Energy drinks (*n* 28)	Total	131 714		156181993		Total	9807		7 668 962		Total	6299		10691346	
	Top 5	129 801	99	155270003	99	Top 5	9667	99	7 618 159	99	Top 5	6114	97	10609237	99
	G Fuel	77 571	59	117764256	75	Nos Energy	8628	88	6 319 573	82	Nos Energy	3964	63	7 515 311	70
	Nos Energy	40 216	31	18321982	12	Red Bull	671	7	495 155	6	Red Bull	1114	18	1 952 510	18
	Red Bull	8925	7	14711888	9	Monster Energy	145	1	718 294	9	Rockstar Energy	571	9	521 662	5
	Monster Energy	1921	1	3 951 548	3	Starbucks	127	1	37 772	0	G Fuel	374	6	605 027	6
	Rockstar Energy	1168	1	520 329	0	G Fuel	96	1	47 365	1	Monster Energy	91	1	14 727	0
Processed snacks (*n* 92)	Total	8131		5 596 166		Total	628		601 400		Total	297		542 016	
	Top 5	3907	48	3 397 483	61	Top 5	505	80	528 798	88	Top 5	206	69	521 893	96
	Doritos	1252	15	2 193 176	39	Dole	342	54	245 119	41	Goldfish	89	30	466 191	86
	Nutella	828	10	167 378	3	Kashi	91	14	29 053	5	Doritos	50	17	22 036	4
	Kraft	634	8	305 352	5	Belvita	25	4	690	0	Nutella	39	13	4297	1
	Hot Pockets	601	7	604 092	11	Nutella	24	4	19 021	3	Pringles	16	5	28 062	5
	Goldfish	592	7	127 485	2	Doritos	23	4	234 915	39	Fritos	12	4	1307	0
Soda (*n* 68)	Total	14 346		6 682 002		Total	874		634 031		Total	892		292 893	
	Top 5	9659	67	4 647 951	70	Top 5	713	82	498 190	79	Top 5	710	80	169 962	58
	Mountain Dew	2880	20	1 707 029	26	V8	303	35	37 681	6	V8	373	42	54 493	19
	Pepsi	2249	16	236 494	4	Coke	148	17	190 100	30	Fanta	157	18	15 806	5
	Coke	1804	13	1 878 258	28	Pepsi	112	13	129 838	20	Coke	63	7	9991	3
	Fanta	1500	10	555 292	8	Fanta	85	10	109 792	17	Pepsi	60	7	38 348	13
	V8	1226	9	270 878	4	Sprite	65	7	30 779	5	Sprite	57	6	51 324	18
Restaurants (*n* 43)	Total	16 091		20844136		Total	343		145 880		Total	607		676 724	
	Top 5	11 313	70	9 864 282	47	Top 5	263	77	106 188	73	Top 5	450	74	323 161	48
	Uber Eats	3062	19	3 097 063	15	KFC	145	42	47 368	32	KFC	198	33	109 978	16
	KFC	2647	16	918 424	4	McDonalds	42	12	32 020	22	Seamless	91	15	21 658	3
	Chipotle	2462	15	3 943 267	19	Uber Eats	26	8	3378	2	McDonalds	81	13	148 790	22
	McDonalds	1855	12	1 648 387	8	Chipotle	25	7	21 117	14	Uber Eats	46	8	19 482	3
	Taco Bell	1287	8	257 141	1	Seamless	25	7	2305	2	Home Chef	34	6	23 253	3

**Fig. 2:**
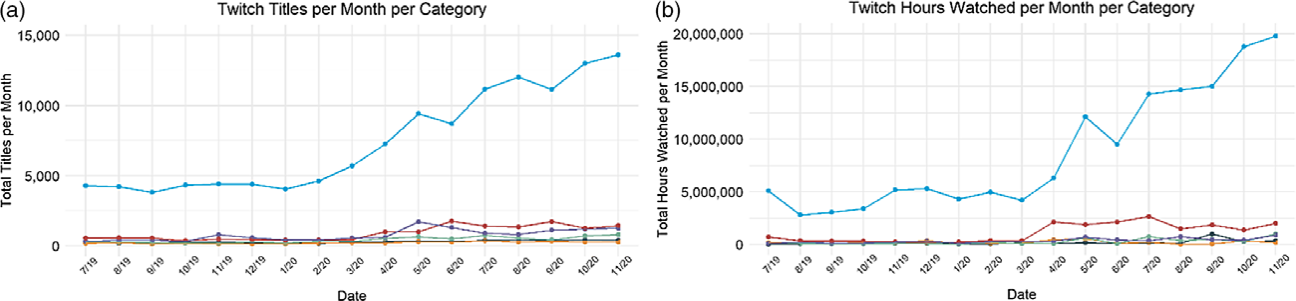
(a) Linear trends of brands mentioned in stream titles across six food and beverage categories on Twitch shown monthly for July 2019–November 2020. (b) Linear trends of hours watched across categories on Twitch shown monthly for July 2019–November 2020. 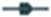, alcohol; 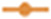, candy; 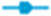, energy drinks; 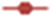, restaurants; 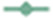, snacks; 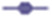, soda

**Fig. 3:**
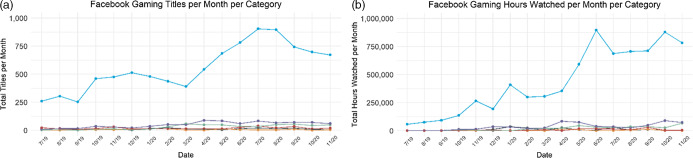
(a) Linear trends of brands mentioned in stream titles across six food and beverage categories on Facebook Gaming shown monthly for July 2019–November 2020. (b) Linear trends of hours watched across categories on Facebook Gaming shown monthly for July 2019–November 2020. 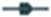, alcohol; 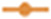, candy; 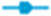, energy drinks; 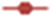, restaurants; 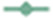, snacks; 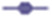, soda

**Fig. 4:**
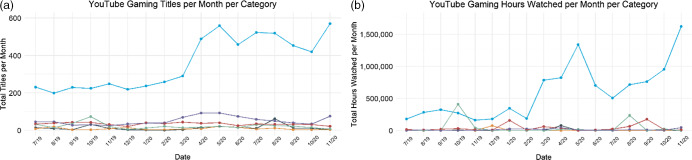
(a) Linear trends of brands mentioned in stream titles across six food and beverage categories on YouTube Gaming shown monthly for July 2019–November 2020. (b) Linear trends of hours watched across categories on YouTube Gaming shown monthly for July 2019–November 2020. 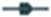, alcohol; 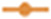, candy; 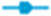, energy drinks; 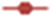, restaurants; 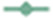, snacks; 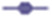, soda

### Twitch

Food and beverage brands or products were mentioned in 179 711 stream titles on the Twitch platform. The predominant category observed in stream titles on Twitch was energy drinks (73 %, *n* 131 174) followed by restaurants (9 %, *n* 16 091), soda (8 %, *n* 14 346), processed snacks (5 %, *n* 8131), alcohol (3 %, *n* 5180) and candy (2 %, *n* 4249). Stream titles containing food and beverage brands or products were viewed for 196 645 623 h. The predominant contributor to hours watched within Twitch was energy drinks (79 %, *n* 156 181 993 h) followed by restaurants (11 % *n* 20 844 136), soda (3 %, *n* 6 682 002), processed snacks (3 %, *n* 5 596 166), alcohol (2 %, *n* 3 443 472) and candy (2 %, *n* 3 897 854). There was a significant increase in the number of brands mentioned in stream titles in each brand category examined on Twitch during the study period (see Fig. 2): soda (289 % increase; *β* = 7·14, 95 % CI 4·82, 9·47, *P* < 0·001), energy drinks (218 % increase; *β* = 67·67, 95 % CI 54·34, 81·00, *P* < 0·001), restaurants (158 % increase; *β* = 9·10, 95 % CI 6·81, 11·40, *P* < 0·001), snacks (148 % increase; *β* = 3·37, 95 % CI 2·19, 4·54, *P* = 0·005), alcohol (77 % increase; *β* = 1·50, 95 % CI 0·97, 2·03, *P* < 0·001) and candy (47 % increase; *β* = 1·13, 95 % CI 0·73, 1·53, *P* < 0·001). For hours watched, there was a significant increase in the hours of brand exposure on Twitch during the study period for snacks (1081 % increase; *β* = 4364·00, 95 % CI 2302·43, 6424·98, *P* < 0·001), soda (719 % increase; *β* = 3712·00, 95 % CI 2196·08, 5227·32, *P* < 0·001), energy drinks (289 % increase; *β* = 106 900, 95 % CI 83 023·03, 130 834·90, *P* < 0·001) and restaurants (171 % increase; *β* = 14 220·00, 95 % CI 8640·19, 19 797·20, *P* < 0·001). No increase was observed for the candy (*P* = 0·87) or alcohol (*P* = 0·06).

### Facebook Gaming

Food and beverage brands or products were mentioned in 11 971 stream titles on the Facebook Gaming platform. The predominant category mentioned on stream titles within Facebook Gaming was energy drinks (82 %, *n* 9807) followed by soda (7 %, *n* 874), processed snacks (5 %, *n* 628), restaurants (3 %, *n* 343), alcohol (2 %, *n* 202) and candy (1 %, *n* 117). Stream titles containing food and beverage brands or products were viewed for 9 275 099 h. The predominant contributor to hours watched within Facebook Gaming was energy drinks (83 %, *n* 7 668 962 h) followed by soda (7 %, *n* 634 031), processed snacks (6 %, *n* 601 400), restaurants (2 %, *n* 145 880), alcohol (2 %, *n* 188 065) and candy (< 1 %, *n* 36 761). There was a significant increase in the number of brands mentioned in stream titles for (see Fig. 3): snacks (1125 % increase; *β* = 4·88, 95 % CI 2·58, 7·17, *P* = 0·003), soda (578 % increase; *β* = 0·37, 95 % CI 1·75, 0·57, *P* = 0·001), energy drinks (157 % increase; *β* = 3·65, 95 % CI 2·60, 4·70, *P* < 0·001) and restaurants (117 % increase; *β* = 0·09, 95 % CI 0·003, 0·17, *P* = 0·04). No increase was observed for candy (*P* = 0·92) or alcohol (*P* = 0·30). There was a statistically significant increase in the hours of brand exposure in soda (4072 % increase; *β* = 347·00, 95 % CI 89·71, 604·26, *P* = 0·01), snacks (3124 % increase; *β* = 321·40, 95 % CI 202·86, 439·99, *P* < 0·001), energy drinks (1270 % increase; *β* = 5470, 95 % CI 4317·42, 6622·62, *P* < 0·001) and restaurants (182 % increase; *β* = 101·70, 95 % CI 37·73, 165·63, *P* = 0·004). No increase was observed for candy (*P* = 0·36) or alcohol (*P =* 0·95).

### YouTube Gaming

Food and beverage brands or products were mentioned in 8434 stream titles on the YouTube Gaming platform. The predominant category mentioned in stream titles on YouTube Gaming was energy drinks (75 %, *n* 6299) followed by soda (11 %, *n* 892), restaurants (7 %, *n* 607), processed snacks (4 %, *n* 297), alcohol (2 %, *n* 186) and candy (2 %, *n* 153). Stream titles containing food and beverage brands or products were viewed for 12 463 262 h. The predominant contributor to hours watched within YouTube Gaming was energy drinks (86 %, *n* 10 691 346 h) followed by restaurants (5 %, *n* 676 724), processed snacks (4 %, *n* 542 016), soda (2 %, *n* 292 893), alcohol (1 %, *n* 119 243) and candy (1 %, *n* 141 040). Energy drinks were the only product category with a significant increase in the number of brands mentioned in stream titles during the study period (see Fig. 4, 246 % increase; *β* = 2·44, 95 % CI 1·68, 3·20, *P* < 0·001). Similarly, only energy drinks experienced a significant increase in the hours of brand exposure on YouTube Gaming during the study window (790 % increase; *β* = 6105·00, 95 % CI 2822·98, 9386·52, *P* = 0·001).

### Adjusted hours per title

**Fig. 5:**
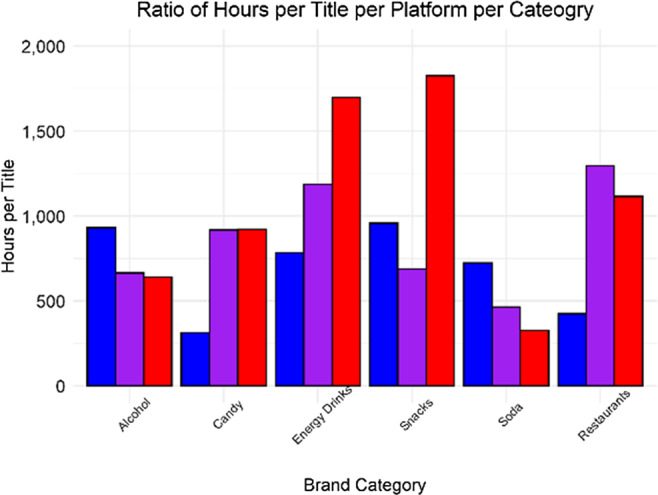
Bar plot depicting the computed ratio of: hours of exposure to a stream title to stream titles containing a food brand or product name for the platforms Twitch, Facebook Gaming and YouTube Gaming across six food and beverage categories. 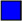, Facebook; 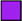, Twitch; 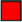, YouTube

There was a significant difference in the ratio of hours watched per titles across brand category by platform (*χ*^2^ = 1514·4, df = 10, *P* < 0·001, Fig. 5). YouTube Gaming had the highest ratio of hours watched per titles (6528 h per title), followed by Twitch (5217 h per title) and Facebook Gaming (4136 h per title). While Twitch had the highest number of food and beverage brands used in stream titles, as well as the highest hours watched, the only category in which Twitch had the highest ratio of hours watched per titles was for restaurants. YouTube Gaming had the highest ratio for candy, energy drinks and snack, while Facebook Gaming had the highest ratio for alcohol and soda.

### Growth pre- and peri-COVID-19 (March 2020)

Collapsed across all brand categories, the interrupted time series analysis suggested a significant shift in number of stream titles containing food brand or product names immediately and following March of 2020 (*β*_pre_ = 0·88, 95 % CI –8·94, 10·71, *P* = 0·85; *β*_immediate_ = 3269, 95 % CI 1341·94, 5195·90, *P* = 0·003; *β*_post_ = 36·01, 95 % CI 23·19, 48·83, *P* < 0·001), as well as hours watched for the Twitch platform (*β*_pre_ = 5196, 95 % CI –14635·39, 25 028·27, *P* = 0·58; *β*_immediate_ = 1 913 411, 95 % CI –1976273·90, 5 803 094·94, *P* = 0·31; *β*_post_ = 61 531, 95 % CI 35 658·70, 87 403·68, *P* < 0·001). On Facebook Gaming, stream titles significantly increased prior to March 2020, but did not significantly increase after this date (*β*_pre_ = 1·50, 95 % CI 0·11, 2·88, *P* = 0·04; *β*_immediate_ = 63·85, 95 % CI –207·85, 335·56, *P* = 0·62; *β*_post_ = –0·51, 95 % CI –2·31, 1·30, *P* = 0·56). This phenomenon was also true of hours watched (*β*_pre_ = 1828, 95 % CI 623·69, 3032·21, *P* = 0·006; *β*_immediate_ = 50 140, 95 % CI –186053·90, 286 337·22, *P* = 0·65; *β*_post_ = 2286, 95 % CI –1342·44, 1799·70, *P* = 0·76). On YouTube Gaming, an immediate increase in stream titles was observed in March 2020, but differences between growth before and after this date were NS (*β*_pre_ =–0·08, 95 % CI –0·98, 0·83, *P* = 0·86; *β*_immediate_ = 263·76, 95 % CI 86·98, 440·54, *P* = 0·007; *β*_post_ = 0·23, 95 % CI –0·94, 1·41, *P* = 0·68). There were no significant differences in hours watched pre-, peri- or post-March 2020 (*β*_pre_ = 197·8, 95 % CI –2855·81, 3251·49, *P* = 0·89; *β*_immediate_ = 453 035·1, 95 % CI –145887·98, 1 051 958·15, *P* = 0·13; *β*_post_ = 1337, 95 % CI –2646·78, 5320·77, *P* = 0·48).

#### The impact of COVID-19 on Twitch food and beverage brand exposure

**Fig. 6:**
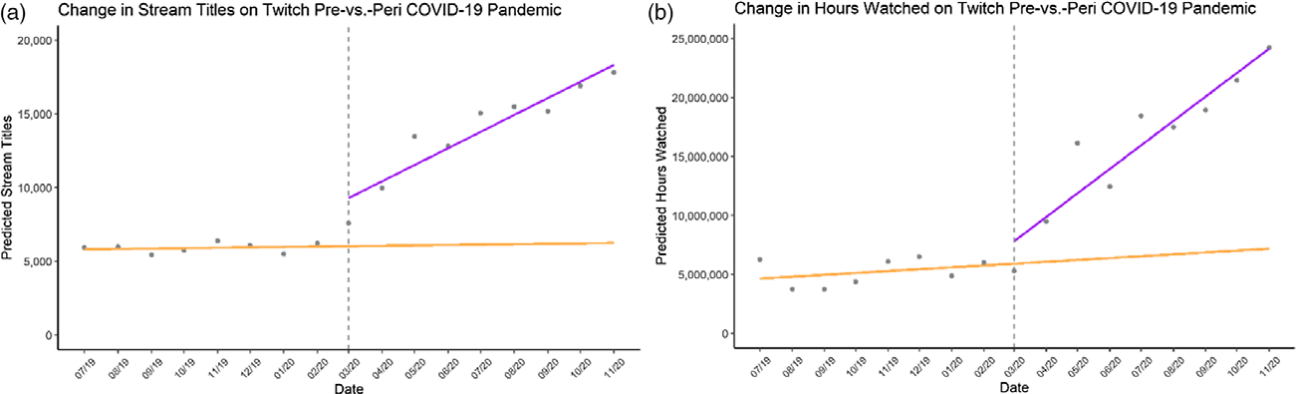
(a) Growth trends for the number of food and beverage brand names used in stream titles across six food and beverage categories on the Twitch Platform pre-March 2020 (yellow line) *v*. after March 2020 (purple line). (b) Growth trends for hours watched on Twitch pre-March 2020 (yellow line) *v*. after March 2020 (purple line). Note that the yellow line post-March 2020 is a projection of the trend had the COVID-19 pandemic not occurred and is not reflective of the true values

Between August 2019 and March 2020, the increase in titles containing energy drink brands on Twitch was NS (*β*_pre_ = 1·32, 95 % CI –5·58, 8·21, *P* = 0·69). The rapid shift to an online environment brought on by COVID-19 introduced an immediate increase in number of titles containing energy drink brands (*β*_immediate_ = 2053·23, 95 % CI 701·10, 8·21, *P* = 0·006) that was sustained in the following months (*β*_post_ = 29·03, 95 % CI 20·03, 3405·37, *P* < 0·001, Fig. 6). Similar results were observed for hours watched of energy drinks (*β*_pre_ = 5934, 95 % CI –9014·47, 20881·90, *P* = 0·41; *β*_immediate_ = 298 656, 95 % CI –2633182·70, 34736·37, *P* = 0·829; *β*_post_ = 54 238, 95 % CI 34 736·37, 73738·99, *P* < 0·001). COVID-19 also impacted stream titles in all other categories: alcohol (*β*_pre_ = –0·21, 95 % CI –0·65, 0·23, *P* = 0·32; *β*_immediate_ = 110·04, 95 % CI 24·02, 196·06, *P* = 0·02; *β*_post_ = 0·73, 95 % CI 0·16, 1·30, *P* = 0·02), candy (*β*_pre_ = –0·12, 95 % CI –0·55, 0·32, *P* = 0·56; *β*_immediate_ =95·49, 95 % CI 10·12, 180·86, *P* = 0·03; *β*_post_ = 0·34, 95 % CI –0·22, 0·91, *P* = 0·21), restaurants (*β*_pre_ = –0·80, 95 % CI –3·48, 1·87, *P* = 0·53; *β*_immediate_ = 518·17, 95 % CI –6·30, 1042·64, *P* = 0·05; *β*_post_ = 3·94, 95 % CI 0·45, 7·42, *P* = 0·03) and processed snacks (*β*_pre_ = –0·04, 95 % CI –1·16, 1·08, *P* = 0·93; *β*_immediate_ = 197·76, 95 % CI –21·79, 417·30, *P* = 0·07; *β*_post_ = 1·10, 95 % CI –0·36, 2·60, *P* = 0·13). COVID-19 impacted hours watched for restaurants (*β*_pre_ = –1402, 95 % CI –6882·15, 4078·69, *P* = 0·59; *β*_immediate_ = 1 382 893, 95 % CI 307 999·85, 2457787·04, *P* = 0·02; *β*_post_ = 3328, 95 % CI –3821·40, 10478·05, *P* = 0·33), but did not have an impact on the hours watched for alcohol, candy or processed snacks, or soda (all *P*’s > 0·05).

#### The impact of COVID-19 on Facebook Gaming food and beverage brand exposure

Prior to March 2020, the use of energy drink brand names in stream titles did not significantly increase (*β*_pre_ = 1·13, 95 % CI –0·17, 2·43, *P* = 0·05). COVID-19 had an immediate effect on energy drink brand names in stream titles (*β*_immediate_ = 27·12, 95 % CI –227·04, 282·10, *P* = 0·005), but the following growth was not statistically significant (*β*_post_ = 0·10, 95 % CI –1·83, 1·55, *P* = 0·08). Hours watched of energy drinks were significantly increasing prior to March 2020 but not after (*β*_pre_ = 1473, 95 % CI 258·14, 2687·94, *P* = 0·02; *β*_immediate_ = 29 380, 95 % CI –208897·08, 267 666·81, *P* = 0·79; *β*_post_ = 449·3, 95 % CI –1135·65, 2037·24, *P* = 0·55). COVID-19 also impacted stream titles in other categories: alcohol (*β*_pre_ = 0·08, 95 % CI 0·02, 0·14, *P* = 0·01; *β*_immediate_ = –7·12, 95 % CI –18·65, 4·41, *P* = 0·21; *β*_post_ = –0·08, 95 % CI –0·16, 0·006, *P* = 0·04), candy (*β*_pre_ = 0·06, 95 % CI 0·02, 0·09, *P* = 0·003; *β*_immediate_ = –9·56, 95 % CI –16·45, 2·66, *P* = 0·01; *β*_post_ =–0·06, 95 % CI –0·10, –0·01 *P* = 0·01), processed snacks (*β*_pre_ = 0·09, 95 % CI –0·0006, 0·17, *P* = 0·05; *β*_immediate_ = 2·71, 95 % CI 9·98, 44·26, *P* = 0·005; *β*_post_ =–1·01, 95 % CI –0·22, 0·13, *P* = 0·08) and soda (*β*_pre_ = 0·16, 95 % CI 0·03, 0·29, *P* = 0·02; *β*_immediate_ =23·62, 95 % CI –1·04, 48·29, *P* = 0·06; *β*_post_ = –0·18, 95 % CI –1·04, 48·29, *P* = 0·04). COVID-19 did not have an impact on the use of restaurant names in stream titles (*P* > 0·05). COVID-19 did not have an impact on the hours watched for alcohol, candy, processed snacks, restaurants or soda (all *P* > 0·05).

#### The impact of COVID-19 on YouTube Gaming food and beverage brand exposure

COVID-19 had an immediate effect on the presence of energy drink (*β*_pre_ = 0·15, 95 % CI –0·53, 0·83, *P* = 0·64; *β*_immediate_ = 173·84, 95 % CI 40·79, 306·90, *P* = 0·02; *β*_post_ = 0·26, 95 % CI –0·62, 1·15, *P* = 0·53) and soda (*β*_pre_ = –0·02, 95 % CI –0·18, 0·13, *P* = 0·76; *β*_immediate_ = 51·33, 95 % CI 20·57, 82·09, *P* = 0·003; *β*_post_ = –0·13, 95 % CI –0·34, 0·07, *P* = 0·19) brand names in stream titles. COVID-19 did not have an impact on the use of alcohol, candy, restaurants or processed snack brands mentioned in stream titles (all *P*’s > 0·05). COVID-19 did not have an impact on the hours watched for any brand category on YouTube Gaming (all *P*’s > 0·05).

## Discussion

This study expands our previously published work on the platform Twitch and describes the presence of, and potential differences between, food and beverage brand exposure on three popular livestreaming sites (Twitch, Facebook Gaming and YouTube Gaming) across six distinct categories (alcohol, candy, energy drinks, processed snacks, soda and restaurants) during a time period of 17 months (July 2019–November 2020). Of these platforms, Twitch was responsible for generating the greatest number of stream titles containing mentions of food and beverage brands and products. Additionally, Twitch was responsible for the majority of hours viewed under these food and beverage brands. This is not surprising given that Twitch is currently the largest livestreaming platform and has structured programmes to drive advertising on their platforms^(11,22)^. Recent work highlights that self-reported exposure to digital advertising for restaurants and food delivery services has been associated with an increased risk of developing obesity^(32)^. Therefore, characterising the prevalence and growth of food and beverage marketing on livestreaming platforms is an important step in understanding the link between food and beverage marketing and rising rates of overweight and obesity.

While the scale of growth between platforms was different in absolute terms, increases were observed in food brand exposure across all platforms. Although we note that growth on Facebook Gaming and YouTube Gaming was found most notably among energy drinks, increases were also observed for restaurants and sodas. These findings extend our previous work and demonstrate that the livestreaming environment, regardless of platform, is heavily comprised of food and beverage marketing and continuing to grow. Across platforms, energy drinks were the most commonly marketed food and beverage product. Energy drinks contain not only high levels of caffeine but also sugar, ingredients with stimulating properties and under-studied novel ingredients^(33,34)^. Energy drinks are packaged in 8–32 oz containers and may contain between 80 and 140 mg of caffeine per 8 oz serving, making it very easy for an adult to reach the upper limit recommendation of daily caffeine intake of 400 mg/d^(35)^. Important to note is that for children and adolescents, an upper limit of 100 mg/d has been proposed, which is met with one serving of many popular energy drinks^(35,36)^. Recent work has also linked video game usage to greater energy drink intake^(37)^, and links have been made between the high caffeine levels in energy drinks and self-reported negative physiological health effects among adolescents and adults, including caffeine toxicity, caffeine dependence and caffeine withdrawal^(37,38)^. The dominance of energy drinks was driven by two brands in particular: G FUEL (which accounted for 59 % of all energy drink titles on Twitch) and NOS Energy (which accounted for 88 % of all energy drink titles on Facebook Gaming and 63 % of all energy drink titles on YouTube Gaming, respectively). While these numbers may represent a high global presence of energy drink marketing, they may also be specific to targeted ad campaigns and sponsored streams. For example, G FUEL, the self-proclaimed ‘official energy drink of esports’, ran one of the most successful ad campaigns on the Twitch platform in 2020, drawing in over 524 000 h of viewership by teaming up with a single top streamer^(11,39)^. To better understand which livestreaming platform provided the greatest exposure per title, we examined a ratio of hours watched per number of stream titles on a given platform. This gives some specific insight into the potential impact of a single stream title on a specific platform despite disparities between viewer counts across platforms. For example, Twitch has substantially more users than the other platforms, but it also has more streamers. As a result, this could dilute the potential impact of a single sponsored stream on the platform. Even though Facebook Gaming and YouTube Gaming have fewer overall viewers, our data suggest that sponsored streams on these platforms may still have a significant impact on generating exposure for brands when key streamers are utilised to promote said brand. Future work is needed to fully characterise the potential differences between demographic characteristics of viewers across platforms to fully understand the differences in marketing on different livestreaming platforms.

Our results also demonstrate that COVID-19 had a significant immediate and sustained impact on the presence of brand exposure on livestreaming platforms. This is in line with reports from marketing research that indicates a large shift of marketing dollars to online platforms in response to COVID-19^(26)^. Indeed, there seems to be some agreement that advertising on digital platforms (including novel technologies such as livestreaming) is likely to continue to grow at a faster rate than previously expected^(26)^. Time spent online had already been labelled ‘unprecedented’, but shelter-in-place orders and the transition to online-based work and school pushed hours spent online to record peaks^(40)^. Livestreaming has traditionally been considered a niche market for video gamers, yet rapid technological shifts have facilitated the movement of daily events to livestreaming: children have begun livestreaming school and social events like virtual birthday parties^(40)^, political figures have livestreamed video games as well as historic events^(41)^ and reality TV, sports and competition shows began trending across livestreaming platforms due to restrictions placed on in-person filming^(42)^. Livestreaming is becoming increasingly commonplace, and according to these findings as well as our previous work, food and beverage marketing may continue to grow as well^(6)^.

While we were able to highlight the massive amounts of brand exposure and corresponding rapid growth on livestreaming platforms, future work is needed to understand the sustained effect this will have on both future marketing strategies and eating behaviours. The role of regulation of marketing on these platforms has been of debate, particularly as it pertains to more vulnerable populations such as children. Worldwide regulations towards food marketing practices vary greatly. For example, in the United Kingdom, government has taken steps towards limiting food and beverage exposure towards children, while any regulation in the USA is essentially non-existent^(43)^. Regulations towards marketing unfortunately lag far behind new forms of technology and media, and those that do exist are aimed towards television advertisements, despite research that suggests that this is becoming a decreasingly relevant form of marketing^(44)^. While data surrounding viewership demographics of Facebook Gaming and YouTube Gaming are limited, nearly half of all Twitch accounts belong to users between the ages of 18–34, and 21 % belong to individuals aged 13–17 years^(45)^. However, when creating an account users self-report their age with no verification, so it is possible that a large percentage of viewers is younger than these reported statistics. Additionally, livestreaming on many ‘mature’ platforms can be viewed without needing an active account by simply passing a self-reported age-check question. Even when disregarding these issues, the high prevalence of young viewership is concerning, as evidence suggests that children are not fully aware of the persuasive intent of marketing and tend to accept advertising as truthful, accurate and unbiased^(46,47)^. The evidence base on the impact of advertising exposure among youth is large and growing, with strong findings linking exposure to changes in consumption^(48–50)^. Additionally, this work is the first to highlight the large prevalence of brand exposure for alcohol on livestreaming platforms. Evidence suggests that alcohol advertising and promotion may increase the likelihood that adolescents will start to use alcohol, and that those who begin drinking as an adolescent are more likely to experience alcohol dependence later in life^(51,52)^. Therefore, present work highlights a need to include new forms of media and marketing (including livestreaming) in future conversations regarding regulation of food, beverage and alcohol marketing and to more fully examine the effects of marketing on adolescents, which has been a traditionally understudied group in the food marketing literature^(53)^.

This study drew its strengths from its novel comparison of brand exposure across multiple popular livestream platforms, yet it is not without limitations. First, we were only able to analyse hours watched as they related to stream titles, while previous work allowed us a more comprehensive view of the advertising on the entire platform (such as streamer profiles). While stream titles and hours watched are able to capture a broad view of brand exposure, livestreaming platforms are abundant with other types of simultaneous brand exposure such as video ads, banner ads, overlay ads, product placements and promotional giveaways, to name a few. Therefore, companies have the ability to simultaneously advertise in a number of different ways on livestreaming platforms, which then may spread onto other social media accounts managed by the streamer and their team. These multiple types of advertising and their potential widespread reach may work synergistically to increase brand recall, positive brand attitude and actual purchasing or food choice behaviour, and future work is needed to fully explore these potential synergistic relationships. Therefore, the results presented here are likely a conservative estimate of the true amount of brand exposure on these platforms as we only accounted for the use of food and beverage brands or products in stream titles, and did not account for other potential accompanying marketing tactics. Second, our analysis was not restricted to including the disclosure of an advertising campaign through the use of hashtag (#) indicators such as ‘#ad’ or ‘#sponsored’. This was a conscious choice, as limiting the search to hashtag indicators would have caused us to miss naturally occurring brand exposure and potential overlap with traditional marketing campaigns (e.g. users livestreaming themselves playing branded mini-games even though the streamer is not sponsored by the brand). Further, our main interest was to comprehensively measure the amount of exposure of food and beverage brands, which takes place regardless of the presence of #ad. Finally, the brands of interest were developed from a published list of brands that advertised on Twitch and was updated by looking at the profiles and streams of the top 100 streamers on Twitch, Facebook Gaming and YouTube Gaming. As a result, brands that were not on the list or began advertising after our search period could have been missed. However, given the extensive list of brands included in the search, the number of brands missed is likely minimal.

This work highlights the continued high levels of exposure to food and beverage brands and products on the livestreaming platforms Twitch, Facebook Gaming and YouTube Gaming, and found that most of the brand exposure was derived from energy drink marketing. It is also the first study to model the real-world impact of the COVID-19 pandemic on food and beverage brand marketing growth on livestreaming platforms. These findings indicate that food and beverage marketing on livestreaming platforms is an important component of online marketing practices and should continue to be monitored.

## Supplementary material

For supplementary material accompanying this paper visit https://doi.org/10.1017/S1368980021004420. click here to view supplementary material
