# Supplementary material for: Prevalence and comparisons of alcohol, candy, energy drink, snack, soda, and restaurant brand and product marketing on Twitch, Facebook Gaming and YouTube Gaming
Source: Public Health Nutr. 2021 Oct 25;25(1):1–12. doi: 10.1017/S1368980021004420 (PMC8593406; doi:10.1017/S1368980021004420)
Supplement: Supplementary file 1 [file S1368980021004420sup.zip › S1368980021004420sup001.docx]

**Supplementary Figures**

| 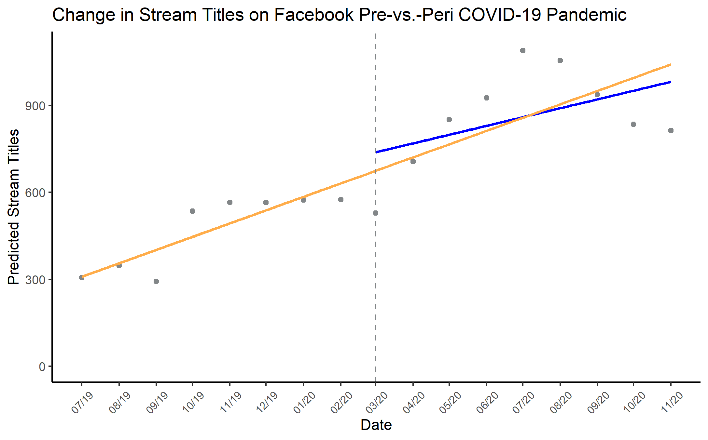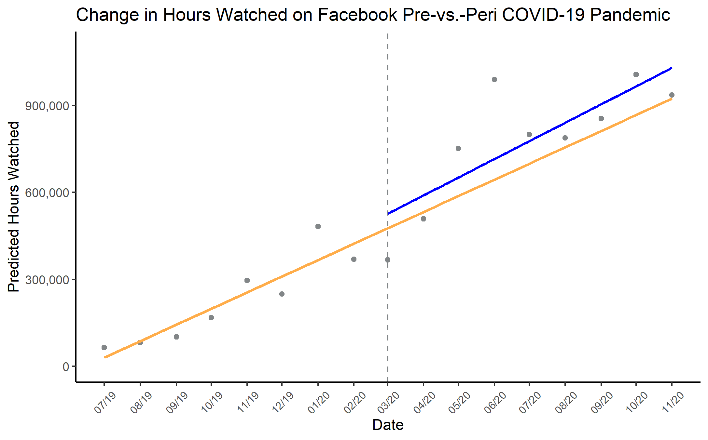  **a)**  **b)** |
| --- |
| **Supplementary Figure 1**. a) Growth trends for the number of food and beverage brand name used in stream titles on the Facebook Gaming Platform pre-March 2020 (yellow line) vs. after March 2020 (blue line) b) Growth trends for hours watched on Facebook Gaming pre-March 2020, after March 2020. Note that the yellow line post March 2020 is a projection of the trend had the COVID-19 pandemic not occurred and is not reflective of the true values. |

| 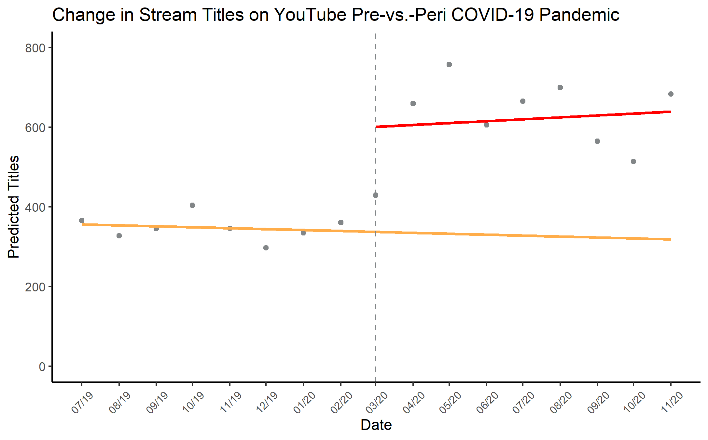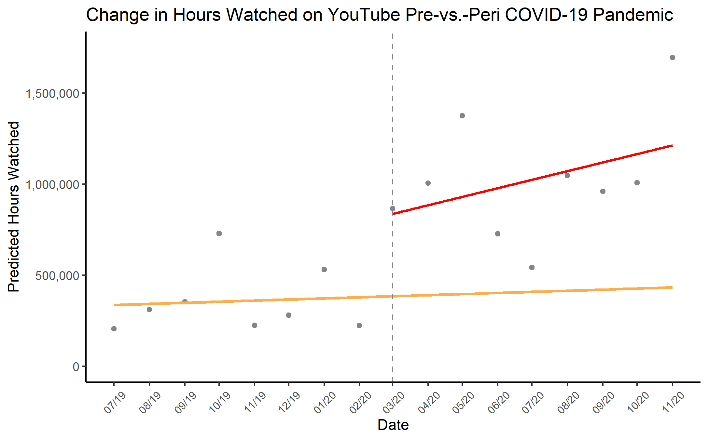  **a)**  **b)** |
| --- |
| **Supplementary Figure 2**. a) Growth trends for the number of food and beverage brand name used in stream titles on the YouTube Gaming Platform pre-March 2020 (yellow line) vs. after March 2020 (blue line) b) Growth trends for hours watched on YouTube Gaming pre-March 2020, after March 2020. Note that the yellow line post March 2020 is a projection of the trend had the COVID-19 pandemic not occurred and is not reflective of the true values. |
