# Supplementary material for: Prevalence and comparisons of alcohol, candy, energy drink, snack, soda, and restaurant brand and product marketing on Twitch, Facebook Gaming and YouTube Gaming
Source: Public Health Nutr. 2021 Oct 25;25(1):1–12. doi: 10.1017/S1368980021004420 (PMC8593406; doi:10.1017/S1368980021004420)
Supplement: Supplementary file 1 [file S1368980021004420sup.zip › S1368980021004420sup002.docx]

Full list of food brands and products searched

| 3 musketeers |
| --- |
| 3 musketeers |
| 3musketeers |
| 5 gum |
| 5 gum |
| 5gum |
| 5hour energy |
| 5-hour energy |
| 5-hour energy |
| 5hourenergy |
| 5-hourenergy |
| 7up |
| 7-up |
| 7-up |
| a&w |
| a&w |
| aandw |
| absolut vodka |
| absolut vodka |
| absolutvodka |
| activia |
| adrenaline rush |
| Adrenaline Rush |
| adrenalinerush |
| air co |
| Air Co |
| airco |
| amd |
| amp energy |
| Amp Energy |
| ampenergy |
| apple & eve |
| apple and eve |
| Apple and Eve |
| apple&eve |
| appleandeve |
| arbys |
| Arbys |
| arby's |
| arctic summer |
| arcticsummer |
| arisona iced tea |
| arisona tea |
| arisonaicedtea |
| arisonatea |
| arizona iced tea |
| Arizona Iced Tea |
| arizona tea |
| arizonaicedtea |
| arizonatea |
| Artic Summer |
| athletic brewing |
| Athletic Brewing |
| athleticbrewing |
| B & G Foods |
| b&g foods |
| b&gfoods |
| baby ruth |
| Baby Ruth |
| babybel |
| babyruth |
| bacardi |
| backwoods |
| bai |
| baileys |
| ballantyne brands |
| Ballantyne Brands |
| ballantynebrands |
| bandg foods |
| bandgfoods |
| bang |
| bard valley |
| Bard Valley |
| bardvalley |
| barefoot |
| barq's |
| bel brands |
| belbrands |
| Belbrands |
| belvedere |
| belvita |
| betty crocker |
| Betty Crocker |
| bettycrocker |
| beyondmeat |
| blue apron |
| Blue Apron |
| blueapron |
| bolthouse farms |
| Bolthouse Farms |
| bolthousefarms |
| bon v!v |
| bon viv |
| Bon Viv |
| bonv!v |
| bonviv |
| boston market |
| Boston Market |
| bostonmarket |
| brisk |
| britvic |
| bsn endorush |
| BSN Endorush |
| bsnendorush |
| bud light |
| bud light lime |
| bud select |
| budlight |
| budlightlime |
| budselect |
| budweiser |
| budweiser |
| bug juice |
| Bug Juice |
| bugjuice |
| burger king |
| Burger King |
| burgerking |
| butterfinger |
| cactus cooler |
| Cactus Cooler |
| cactuscooler |
| campbells |
| canada dry |
| Canada Dry |
| canadadry |
| canadian club |
| Canadian Club |
| canadianclub |
| capri sun |
| Capri Sun |
| caprisun |
| captain morgan |
| Captain Morgan |
| captainmorgan |
| carls jr. |
| carl's jr. |
| Carls Junior |
| carlsjr |
| carl'sjr |
| carlsjr. |
| carl'sjr. |
| cb distributors |
| CB Distributors |
| cbdistributors |
| celsius |
| cheerwine |
| cheesit |
| chees-it |
| cheetos |
| cheezit |
| cheez-it |
| Cheez-It |
| chex |
| chex mix |
| Chex Mix |
| chexmix |
| chickfila |
| chick-fil-a |
| Chick-Fil-A |
| chipolte |
| chipotle |
| Chipotle |
| chips ahoy |
| Chips Ahoy |
| chipsahoy |
| chobani |
| chownow |
| Cici’s pissa |
| Cici’s pizza |
| Cici’spissa |
| Cici’spizza |
| cicis pissa |
| cici's pissa |
| cicis pizza |
| Cicis Pizza |
| cici's pizza |
| cicispissa |
| cici'spissa |
| cicispizza |
| cici'spizza |
| Clif |
| clif bar |
| Clif Bar |
| clifbar |
| cocacola |
| Coke |
| Coke |
| conagra |
| conagra foods |
| Conagra Foods |
| conagrafoods |
| Coors |
| coors banquet |
| coors light |
| coorsbanquet |
| coorslight |
| corona |
| Corona |
| corona extra |
| corona light |
| coronaextra |
| coronalight |
| crook & marker |
| crook and marker |
| Crook and Marker |
| crook&marker |
| crookandmarker |
| crown royal |
| Crown Royal |
| crownroyal |
| dairy queen |
| Dairy Queen |
| dairyqueen |
| danimals |
| dannon |
| Dannon |
| danonino |
| dan-o-nino |
| diamond foods |
| Diamond Foods |
| diamondfoods |
| dieser andere energy drink |
| Diets & Watson |
| Diets & Watson Nuts |
| Diets and Watson |
| Diets and Watson Nuts |
| Diets&Watson |
| Diets&WatsonNuts |
| DietsandWatson |
| DietsandWatsonNuts |
| Dietz & Watson |
| Dietz & Watson Nuts |
| Dietz and Watson |
| Dietz and Watson Nuts |
| Dietz&WatsonNuts |
| DietzandWatson |
| DietzandWatsonNuts |
| Dole |
| dole fruit bowls |
| dolefruitbowls |
| dominos |
| Dominos |
| domino's |
| door dash |
| Door Dash |
| doordash |
| doritos |
| dos equis |
| Dos Equis |
| dosequis |
| dove chocolate |
| Dove Chocolate |
| dovechocolate |
| dr pepper |
| Dr Pepper |
| drpepper |
| dunkin donuts |
| Dunkin Donuts |
| dunkin donut's |
| dunkindonuts |
| dunkindonut's |
| dunkin'donuts |
| Fage |
| Fanta |
| faygo |
| ferrero |
| ferrero rocher |
| ferrero rocher |
| ferrerorocher |
| fiber one |
| Fiber One |
| fiberone |
| Ficks |
| fig newtons |
| Fig Newtons |
| fignewtons |
| Fresh Direct |
| frito lay |
| fritolay |
| Fritos |
| Fritos |
| fruit rush |
| Fruit Rush |
| fruitfulls |
| FruitFulls |
| fruit-fulls |
| fruitrush |
| full throttle |
| Full Throttle |
| fullthrottle |
| g fuel |
| G Fuel |
| game fuel |
| gamefuel |
| gamer fuel |
| gamerfuel |
| gatorade |
| general mills |
| General Mills |
| generalmills |
| Gfuel |
| ghiradelli |
| Glaceau Vitamin Water |
| glaceau vitaminwater |
| glaceauvitaminwater |
| gogo squeese |
| gogo squeeze |
| gogosqueese |
| gogosqueeze |
| gogurt |
| gold peak |
| goldfish |
| goldfish crackers |
| goldfishcrackers |
| goldpeak |
| grand marnier |
| grandmarnier |
| grase snack boxes |
| grasesnackboxes |
| graze snack boxes |
| grazesnackboxes |
| grey goose |
| Grey Goose |
| greygoose |
| grub hub |
| grubhub |
| Grubhub |
| guinness |
| hardees |
| Hardees |
| hardee's |
| Haus |
| hawaiian punch |
| hawaiianpunch |
| heineken |
| hennessy |
| henrys |
| henry's |
| Henry's |
| hersheys |
| hershey's |
| Hershey's |
| hiball |
| Hic |
| hi-c |
| Hi-c |
| high noon |
| High Noon |
| highnoon |
| home chef |
| Home Chef |
| homechef |
| honest tea |
| honesttea |
| honey maid |
| honeymaid |
| hyperice |
| Ibc |
| imperfect foods |
| imperfectfoods |
| impossible foods |
| impossiblefoods |
| instacart |
| jack daniels |
| jack daniel's |
| Jack Daniel's |
| Jack in the Box |
| jack links |
| jackdaniels |
| jackdaniel's |
| jackinthebox |
| jack-in-the-box |
| jarritos |
| java monster |
| javamonster |
| Jello |
| jersey mikes |
| jersey mike's |
| jerseymikes |
| jerseymike's |
| Jersy Mikes |
| Jif |
| Jif |
| Jiff |
| jim beam |
| Jim Beam |
| jimbeam |
| jm smucker |
| jmsmucker |
| johanna foods |
| johannafoods |
| jones soda |
| jonessoda |
| jose cuervo |
| Jose Cuervo |
| josecuervo |
| jumex |
| june shine |
| June Shine |
| juneshine |
| Kashi |
| keebler |
| kellogg |
| Kellogg |
| kelloggs |
| kellogg's |
| ketel one |
| ketelone |
| keurig |
| Kfc |
| Kind |
| kings coast cofee company |
| kings coast coffee |
| kingscoastcofeecompany |
| kingscoastcoffee |
| kit kat |
| Kit Kat |
| kitkat |
| kliff cliff |
| kliffcliff |
| kodiak |
| Kool Aid |
| koolaid |
| kool-aid |
| kosy shack |
| kosy shak |
| kosyshack |
| kosyshak |
| kozy shack |
| kozy shak |
| kozyshack |
| kozyshak |
| Kraft |
| langers |
| laughing cow |
| laughingcow |
| Lays |
| Lays |
| lay's |
| link snacks |
| linksnacks |
| lipton |
| little caesars |
| little debbie |
| little hug |
| little hug fruit barrels |
| littlecaesars |
| littledebbie |
| littlehug |
| littlehugfruitbarrels |
| long john silvers |
| long john silver's |
| longjohnsilvers |
| longjohnsilver's |
| m&ms |
| m&ms |
| m&m's |
| madrinas coffee |
| madrinascoffee |
| malibu rum |
| maliburum |
| mandms |
| mandm's |
| mansita sol |
| mansitasol |
| manzita sol |
| manzitasol |
| materne |
| Mcdonald’s |
| Mcdonalds |
| McDonalds |
| mcdonald's |
| Mcds |
| mcd's |
| Mckee |
| mckee foods |
| mckeefoods |
| mello yello |
| Michelob |
| mighty swell |
| mightyswell |
| mikes hard lemonade |
| Mikes Hard Lemonade |
| mike's hard lemonade |
| mikeshardlemonade |
| mike'shardlemonade |
| milky way |
| Milky Way |
| Milkyway |
| miller chill |
| miller high life |
| miller lite |
| Miller Lite |
| Millerchill |
| millerhighlife |
| Millerlite |
| Milos |
| Milos |
| milo's |
| minute maid |
| Minute Maid |
| minutemaid |
| mixyourcandy |
| Modelo |
| Mondeles |
| Mondelez |
| mondo fruit squeesers |
| mondo fruit squeezers |
| mondo squeesers |
| mondo squeezers |
| mondofruitsqueesers |
| mondofruitsqueezers |
| mondosqueesers |
| mondosqueezers |
| Monster |
| monster energy |
| Monster Energy |
| monsterenergy |
| Mott’s |
| Motts |
| mott's |
| mountain dew |
| Mountain Dew |
| mountain dew (game fuel) |
| mountaindew |
| mountaindew(gamefuel) |
| mtn dew |
| Mtndew |
| Munchies |
| nathans hotdogs |
| Nathans Hotdogs |
| nathan's hotdogs |
| nathanshotdogs |
| nathan'shotdogs |
| national atlantic trading co |
| nationalatlantictradingco |
| nature delivered |
| nature valley |
| Nature Valley |
| naturedelivered |
| naturevalley |
| Nestea |
| Nestle |
| Newman’s own |
| Newman’sown |
| Newtons |
| Nos |
| nos energy |
| Nos Energy |
| Nosenergy |
| Novamex |
| Nutella |
| Nutrigrain |
| nutri-grain |
| Oberto |
| ocean spray |
| Ocean Spray |
| oceanspray |
| Oikos |
| Oreo |
| Oreo |
| Oreos |
| oscar mayer |
| oscarmayer |
| pabst blue ribbon |
| pabstblueribbon |
| panera bread |
| panerabread |
| papa johns |
| Papa Johns |
| papa john's |
| Papajohns |
| papajohn's |
| patrÃn |
| Patron |
| peace tea |
| Peacetea |
| Peets |
| Peets |
| peet's |
| pepperidge farm |
| pepperidgefarm |
| Pepsi |
| Pibb |
| pirates booty |
| piratesbooty |
| pissa hut |
| Pissahut |
| pizza hut |
| Pizza Hut |
| Pizzahut |
| Planters |
| planters nuts |
| plantersnuts |
| plum organics |
| plumorganics |
| polar beverages |
| polarbeverages |
| Pom |
| pop tarts |
| Pop Tarts |
| Popchips |
| Popeyes |
| Poptarts |
| Postmates |
| Powerade |
| Press |
| Pringles |
| procter & gamble |
| procter and gamble |
| procter&gamble |
| procterandgamble |
| pura still |
| Purastill |
| pure leaf |
| Pureleaf |
| Quaker |
| quaker snack |
| quakersnack |
| Quisnos |
| Quiznos |
| rc cola |
| RC Cola |
| Rccola |
| red bull |
| Red Bull |
| Redbull |
| Reeses |
| Reeses |
| reese's |
| reeses (take 5) |
| reeses(take5) |
| Reign |
| rice crispies |
| rice krispies |
| Rice Krispies |
| ricecrispies |
| ricekrispies |
| Rits |
| Ritz |
| Ritz |
| Robinsons |
| Robinsons |
| robinson's |
| robinsons fruit shoot |
| robinsonsfruitshoot |
| Rockstar |
| rockstar energy |
| Rockstar Energy |
| rockstarenergy |
| rold gold |
| Roldgold |
| rw knudsen |
| rwknudsen |
| samuel adams boston lager |
| samueladamsbostonlager |
| santa crus organics |
| santa cruz organics |
| santacrusorganics |
| santacruzorganics |
| Sargento |
| Sausa |
| Sauza |
| Saxbys |
| saxby's |
| screamin sicilian |
| screaminsicilian |
| seagramÃ•s |
| Seamless |
| sennheiser |
| Shasta |
| sheila g |
| sheila g brands |
| Sheilag |
| sheilagbrands |
| sierra mist |
| Sierramist |
| Simply |
| Sipfiss |
| sk energy |
| Skenergy |
| Skittles |
| Skoal |
| skyy vodka |
| skyyvodka |
| slim jim |
| Slim Jim |
| Slimjim |
| Smartfood |
| Smirnoff |
| Smucker |
| Smuckers |
| Smuckers |
| Snapple |
| Snickers |
| snow drinks |
| snowdrinks |
| Snyder’s |
| snyders of hanover |
| snyder's of hanover |
| snydersofhanover |
| snyder'sofhanover |
| Sobe |
| Sonic |
| sour patch kids |
| Sour Patch Kids |
| sourpatchkids |
| southern comfort |
| southerncomfort |
| special k |
| Special K |
| Special |
| Sprite |
| Ssips |
| stacker 2 energy |
| stacker 2 xtra |
| stacker2energy |
| stacker2xtra |
| Stacys |
| stacy's |
| stacys pita chips |
| Stacys Pita Chips |
| stacy's pita chips |
| stacyspitachips |
| stacy'spitachips |
| starbucks |
| Starr |
| stella artois |
| stellaartois |
| stewartÃ•s fountain classics |
| stewartÃ•sfountainclassics |
| stolichnaya |
| stormy ginger beer |
| stormygingerbeer |
| Subway |
| sun belt bakery |
| sunbeltbakery |
| sunchips |
| Sundrop |
| Sunkist |
| sunny d |
| sunny delight |
| Sunny Delight |
| Sunnyd |
| sunnydelight |
| sunshine |
| sunsweet |
| sunvalleydairy |
| swedish fish |
| swedishfish |
| sweedish match |
| sweedishmatch |
| sweet leaf |
| Sweet Leaf |
| sweetleaf |
| Swisher |
| swisher international |
| swisherinternational |
| taco bell |
| Taco Bell |
| Tacobell |
| tahitian treat |
| tahitiantreat |
| Tampico |
| Taso |
| Tazo |
| Tazo |
| teddy grahams |
| Teddy Grahams |
| teddygrahams |
| the wonderful company |
| thewonderfulcompany |
| thinkthin |
| thinkthin products |
| thinkthinproducts |
| tootsie rolls |
| tootsierolls |
| tornado energy |
| Tornado Energy |
| tornadoenergy |
| Tostitos |
| Totinos |
| Totinos |
| totino's |
| Triscuit |
| tropicana |
| Truly |
| Tweaker |
| twisslers |
| twisted tea |
| Twisted Tea |
| twistedtea |
| Twix |
| twizzlers |
| two if by tea |
| two lane |
| twoifbytea |
| Twolane |
| Tyson |
| uber eats |
| Uber Eats |
| Ubereats |
| Ubr |
| Uptime |
| v8 |
| V8 |
| v8 fusion |
| v8 splash |
| v8fusion |
| v8splash |
| Vernors |
| vita coco |
| Vitacoco |
| vitamin water |
| Vitamin Water |
| vitaminwater |
| Vpx |
| vuka energy |
| vukaenergy |
| welch foods |
| Welch’s |
| welchfoods |
| Wendys |
| Wendys |
| wendy's |
| west coast chill |
| westcoastchill |
| wheat thins |
| Wheat Thins |
| wheatthins |
| white claw |
| White Claw |
| white owl |
| whiteclaw |
| whiteowl |
| wild basin |
| wildbasin |
| Willies |
| Willies |
| willie's |
| wonderful halos |
| wonderful pistachios |
| wonderfulhalos |
| wonderfulpistachios |
| Xingtea |
| Yoplait |
| york peppermint patties |
| yorkpeppermintpatties |
| Zaxbys |
| Zaxbys |
| zaxby's |
| Zevia |
| Zipfizz |
